# Year of the Brain in Europe 2014 – a new impulse to strengthen the alliance for brain health

**DOI:** 10.3325/cmj.2013.54.417

**Published:** 2013-10

**Authors:** Mary Baker

**Affiliations:** President, European Brain Council

Year of the Brain in Europe 2014 is important and timely because across the region we have an aging population that is increasingly in need of effective care and therapies. But 2014 is also an occasion to celebrate the work of both clinicians and the pharmaceutical industry for providing a standard of health care that enables people to lead a good life.

In the UK a century ago, a man’s life expectancy was just 42 years of age. This has now risen to 78, and in Japan, a little girl born today has a 50-50 chance of living to be 100 years old. However, in the wake of these achievements come big challenges and one of these is that the longer we live, the greater the number of chronic illnesses and brain diseases we will experience. Brain diseases including stroke, Parkinson disease, and Alzheimer disease cause terrible distress if poorly managed, and can represent a considerable and expensive burden for the health care systems.

Furthermore, another growing problem is that the longer we live the more diseases we acquire, so a person over the age of 65 will often have more than three or four diseases at one time, precipitating a situation where comorbidity and polypharmacy will present a major challenge in both the near and distant future.

## Pan-European challenges

We have witnessed unprecedented migration across Europe; time in consultation with a doctor is just 12 minutes; and we are challenged by European states having different degrees of access to medicines. With all these challenges and more, we want to raise awareness in society – not just of doctors, nurses, and politicians – but of ordinary families across Europe. What role can they play in the management of their illnesses as they age and how can they get the best quality of life?

One of the areas where we can obtain measureable results is stroke prevention. How can we educate our publics in our respective countries not just about raised blood pressure and cholesterol, but also about the many other risk factors associated with stroke? How can we prompt people to visit the doctor so that they can access medicines earlier to help prevent stroke?

Other significant issues include how we help raise the awareness of pain. Pain is unseen, it emanates in the brain, it is associated with high costs for labor market, with absenteeism from work, particularly from back pain. What can we do about the psychological and psychiatric diseases – depression, schizophrenia, bipolar disorder – these are all increasing in incidence, and we know that the chance of these people making it to a long life are reduced considerably.

How can we help families educate their children as they are growing up about the challenges of alcohol? We, at the European Brain Council, all believe that alcohol is a greater challenge than drugs because drugs are illegal and there is a very clear warning against their use. Alcohol and tobacco are not illegal, and yet young people are moving relentlessly toward reckless using of these stimulants. We want to raise awareness that we need good science and good clinicians, but also that we want to highlight the importance of economics and ethics – why should some countries in Europe get things that others do not? The voice of the patient is also incredibly important including patients’ experiences about what it is like to live daily with some of these diseases and what it is like to care for somebody with these diseases.

## A series of one-day conferences across Europe

During the Year of the Brain in Europe we want to raise these issues in a series of small one-day conferences across Europe. We do not know all the needs of all the various countries, but as a logo on the conference program already says “doing this with the European Brain Council,” we want to make sure that for one day in 2014 we work together to raise awareness of how the public can actively manage their lives and brain diseases in a better way. The European Brain Council has aimed to bring together European stakeholders from across various specialties related to brain diseases: neurology, psychiatry, neuroscience, neurosurgery, neuropsychopharmacology, patient groups living with neurological illness, and patients groups living with mental illness. Although there are challenges associated with this due to language differences, economic availability of medications, culture, and information, as an organization, we are trying to provide an overarching canopy encompassing individual brain councils devising a program that is optimal for each individual European country. We are trying to organize brain councils in every one of these European countries, and gradually the network of national brain councils will become more extensive.

Patient organizations are also of the utmost importance to the European Brain Council. They will attend many of these conferences and speak about what it is like to live with a certain illness, the challenges of trying to get work, deal with their children or even their grandchildren, manage on transport systems, and to establish what exactly would make a difference to their daily lives. In addition, there is emphasis on how to improve and encourage joint decision making over new therapies, with open discussion of benefit and risk between doctor and patient.

A major objective of the European Brain Council is to engage with society. In collaboration with the World Federation of Neurology, we are in discussion with World Health Organization with the aim of organizing a Day of the Brain, effectively a special day which could be rolled out worldwide to discuss issues related to the brain. As a legacy of the Year of the Brain in Europe, we hope that the European Commission will run a special brain event within the European Parliament. Hopefully, such a meeting could evolve into a meeting similar to that in Davos, where all the world’s economic leaders meet. At that sort of a policy level in the real world, I hope that we might be able to show that we have reduced the number of stroke cases across Europe, because that would have measureable impact.

There are also certain policy-related activities that we aim to do. For example, we aim to encourage all cyclists in Europe to protect their brains by wearing helmets and to help young university students to limit drinking. Also, we would like to see childhood brain diseases receive more attention than that received at the moment. Attention deficit Hyperactivity Disorder (ADHD), autism, and some of rare neuropediatric illnesses benefit from very little research and funding.

In addition, there are also illnesses for which young women take medications from early ages – epilepsy, bipolar disorder, schizophrenia – and yet little is known on how these drugs effect their bodies in the long term and how this manifests in effects on the fetus and uterus during pregnancy. Women have been excluded from the clinical trials for over 50 years because of the thalidomide disaster. What revisions are needed in clinical trials because they exclude most people over the age of 60 and yet the populations most in need of therapies are 60 years plus? How can clinical trials be developed to account for the many co-morbidities older people have? Should there be a new regulatory system? How could we help to improve regulations in Europe, because the one thing we are not short of is wonderful science, but sometimes that science is not underpinned by humanities and ethics?

We also hope that the all these efforts will not just be about curing diseases, but about looking at what the brain has achieved for mankind – the culture, the poetry, the drama, the music, because the brain is responsible for everything that we do. We want not only to encourage more good science, but we also want to ensure some entertainment value. We are planning an event in Brussels about neurological illness, featuring dystonia. One of the main guests will be a concert flautist with dystonia who will play beautiful music illustrating the effect that the disease has on ability to play. We also have a young concert pianist with multiple sclerosis.

Above all, in whichever European country we live in, our mantra is “the national wealth is the brain health.” We want to encourage the youth, who are our future, to develop their brains, nurture their brains, and above all protect their brains. We want to organize events that will educate them in this respect, and hopefully will leave a legacy of a healthier population.

